# Children with Narcolepsy type 1 have increased T‐cell responses to orexins

**DOI:** 10.1002/acn3.50908

**Published:** 2019-11-15

**Authors:** Andrew C. Cogswell, Kiran Maski, Thomas E. Scammell, Dominique Tucker, Zachary S. Orban, Igor J. Koralnik

**Affiliations:** ^1^ Department of Neurological Sciences Rush University Medical Center Chicago Illinois; ^2^ Department of Neurology Boston Children’s Hospital Boston Massachusetts; ^3^ Department of Neurology Beth Israel Deaconess Medical Center Boston Massachusetts

## Abstract

Narcolepsy type 1 (NT1) is caused by severe loss of the orexin neurons, and is highly associated with HLA DQB1*06:02. Using intracellular cytokine staining, we observed a higher frequency of IFN‐γ‐ and TNF‐α‐producing CD4^+^ and CD8^+^ T‐cells in response to orexins in 27 children with NT1 compared to 15 healthy control children. Conversely, no such difference was observed between 14 NT1 and 16 HC adults. In addition, priming with flu peptides amplified the T‐cell response to orexins in children with NT1. Our data suggests that NT1 may be caused by an autoimmune T‐cell response to orexins, possibly triggered by flu antigens.

## Introduction

Narcolepsy type 1 (NT1) is a neurological disorder characterized by chronic daytime sleepiness, cataplexy, hypnagogic hallucinations, and sleep paralysis. NT1 is caused by a selective loss of over 90% of neurons in the lateral hypothalamus that produce orexin‐A and ‐B (also called hypocretin‐1 and −2).^1^, ^2^, ^3^

What causes the death of the orexin neurons remains unclear, but several lines of evidence suggest that NT1 is an autoimmune disease.^4^ HLA‐typing and genome‐wide association studies have shown that >90% of NT1 subjects possess the MHC‐II allele DQB1*06:02,^5^, ^6^ suggesting an immune‐mediated etiology.^7^, ^8^ Occurrence of NT1 in children has been associated with the Pandemrix flu vaccine, raising the concern of molecular mimicry.^4^

Recent reports suggest that both CD4^+^ and CD8^+^ T‐cells from people with NT1 may target the orexin neuropeptides.^9^, ^10^, ^11^ However, whether flu cross‐reactivity is a characteristic of orexin‐specific T‐cells remain a matter of debate.^9^, ^11^, ^12^ In addition, only one of these studies functionally evaluated the cellular immune response^9^ while the others used T‐cell receptor sequencing, X‐Ray crystallography, and peptide prediction software in conjunction with multimer labeling.^10^, ^11^, ^12^ Thus, we characterized the cellular immune response to orexins mediated by CD4^+^ and CD8^+^ T‐cells in peripheral blood of NT1 subjects and determined whether it can be amplified by priming these cells with flu antigen.

## Subjects/Materials and Methods

### Study population

All subjects participating in this study signed informed consent according to the local institutional review board (IRB) guidelines and the guidelines of the IRB of Rush University Medical Center (RUMC). We enrolled a total of 41 NT1 subjects (27 children ages 8–21 and 14 adults ages 25–63) from the Sleep Disorders Clinics at Boston Children’s Hospital and Beth Israel Deaconess Medical Center (BIDMC). To be included in the study, subjects needed to carry MHC DQB1*06:02^+^, have definite cataplexy, and have a Multiple Sleep Latency Test (MSLT) showing short sleep latencies (<8 min) and at least 2 sleep onset rapid eye movement (REM) sleep periods.^13^ The control group consisted of 31 age‐ and sex‐matched healthy control (HC) subjects.

### Determination of T‐cell responses to orexin by Intracellular Cytokine Staining (ICS)

#### Peptide library

The prepro‐orexin protein includes orexin‐A (33aa) and orexin‐B (28 aa) and no other known peptides. Our peptide library included 30 peptides of 15 aa in length, overlapping by 11 aa. In addition, we utilized an Influenza Hemagglutinin1 protein peptide library (BEI) of similar length. With this approach, any 8–11 aa MHC class I epitope recognized by CD8^+^ T‐cells, or any 14 aa MHC class II epitope recognized by CD4^+^ T‐cell epitope could be presented.

We collected up to 30 mL of blood in heparinized tubes depending on age and weight of study subjects, and isolated up to 5 x 10^7^ PBMC by centrifugation over a Ficoll gradient. Cells were then centrifuged, washed, and resuspended in R‐12 (RPMI 1640–12% fetal calf serum) media at a concentration of 3.5/10^6^ cells/mL. For adult subjects, the orexin peptide library was subdivided and pulsed into four equal pools at a final concentration of 2 ug/uL, each spanning an equivalent amount of the protein. Due to the limited number of PBMCs obtained from pediatric subjects, cells were pulsed with the entire orexin peptide library together at a final concentration of 2 ug/uL**.** Cells were incubated in R‐12 medium, and after 72 h, were supplemented with 25 U/mL interleukin‐2 (IL‐2) and incubated for 10 to 14 days prior to performing ICS as previously described.^14^

Briefly, after being counted with an automated cell counter (Countessa, BD), 10^6^ cells were resuspended in 200 uL of R‐12 media containing either flu peptides, orexin peptides, positive controls or no stimuli. All cells were incubated for 1 h at 37°C. Subsequently, 50 uL of diluted 1% monensin (Golgistop; BD Biosciences) was added, followed by incubation for 5 h at 37°C. The cells were then washed and stained with a live/dead exclusion dye (Invitrogen) and surface marker antibodies for CD8 (Biolegend, clone SK1) and CD4 (Biolegend; clone SK3). Subsequently, cells were fixed with 100 L Cytofix/Cytoperm (BD Biosciences) and stained with anti‐human IFN‐γ antibody (Biolegend; clone B27), anti‐human TNF‐α antibody (Biolegend; clone Mab11), and anti‐human CD3 antibody (BD Biosciences; clone UCHT1). Cells were fixed with 1.5% formaldehyde‐PBS. Samples were run on a flow cytometer (LSRFortessa; BD Biosciences) and analyzed, using FlowJo software (BD). Cells were first gated based on forward‐ and side‐scatter to identify lymphocytes, then dead cells were excluded using a live/dead dye. T‐cells were identified as CD3^+^ cells and finally divided into CD4^+^ and CD8^+^ subpopulations. Results were expressed as % cytokine‐producing CD4^+^ or CD8^+^ T‐cells. The ICS results were analyzed after subtraction of the baseline cytokine secretion for the sum of each pool for adults or from the entire library for children for the absolute subject’s response. Negative controls consisted of no peptides (baseline) and a positive control consisted of phorbol 13‐myristate 12‐acetate and ionomycin stimulation of cells.

#### Priming experiments

Similar to the above, PBMCs from a subset of 10 pediatric NT1 and 7 HC subjects were initially pulsed with either the orexin (O) or H1 flu (F) peptide libraries and cultured as previously described. After 10–14 days, cells were rechallenged with either the same peptide library they were initially stimulated with (i.e. O ‐> O, F ‐> F), or the reciprocal peptide library ( i.e. O ‐> F, F ‐> O). IFN‐γ production was then analyzed by ICS as described above.

### Statistical analyses

**Figure 1:**
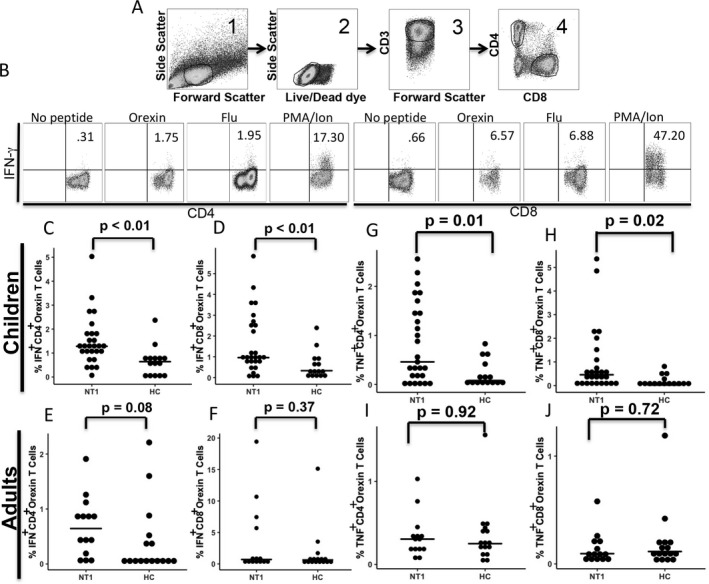
Measurement of T‐cell responses to orexin peptides in peripheral blood. (A). Representative example of gating strategy used in flow cytometry to identify T‐cells from peripheral blood mononuclear cells of one NT1 subject. Cells were gated based on size and granularity (panel 1), living cells (panel 2), CD3^+^ T‐cells (panel 3), and subdivided into CD4^+^ or CD8^+^ (panel 4). (B) Frequency of IFN‐γ‐producing cells, shown in the upper right quadrant of each panel were measured after stimulation with peptide libraries covering orexins or the H1 flu protein as well as no peptide as a negative control (baseline) and PMA/Ionomycin as a positive control.(C,D,E,F) Frequency of CD4^+^ (C,E) and CD8^+^ (D, F) T‐cells‐ producing IFN‐γ quantified by ICS for either pediatric (C, D) or adult (E, F) NT1 subjects and healthy controls (HC) after stimulation with orexin peptide library.(G,H,I,J) Frequency of CD4^+^ (G, I) and CD8^+^ (H, J) T‐cells‐producing TNF‐α quantified by ICS for either children (G, H) or adults (I, J) NT1 subjects and healthy controls (HC) after stimulation with orexin peptide library. The bars indicate the median results for the respective groups. The range and mean for the baseline values of cytokines were as follows: Children CD4+ T‐cells, IFN‐γ: 0.00‐5.84/ 1.19; TNF‐α: 0.37‐2.65/ 0.93. CD8+ T‐cells, IFN‐γ: 0.00‐2.39/ 1.22; TNF‐α: 0.50‐2.20/ 1.69. Adult CD4+ T‐cells, IFN‐γ: 0.01‐0.55/ 0.13; TNF‐α: 0.02‐0.25/ 0.08. CD8+ T‐cells, IFN‐γ: 0.08‐3.78/ 0.63; TNF‐α: 0.00‐0.17/ 0.04.

**Figure 2:**
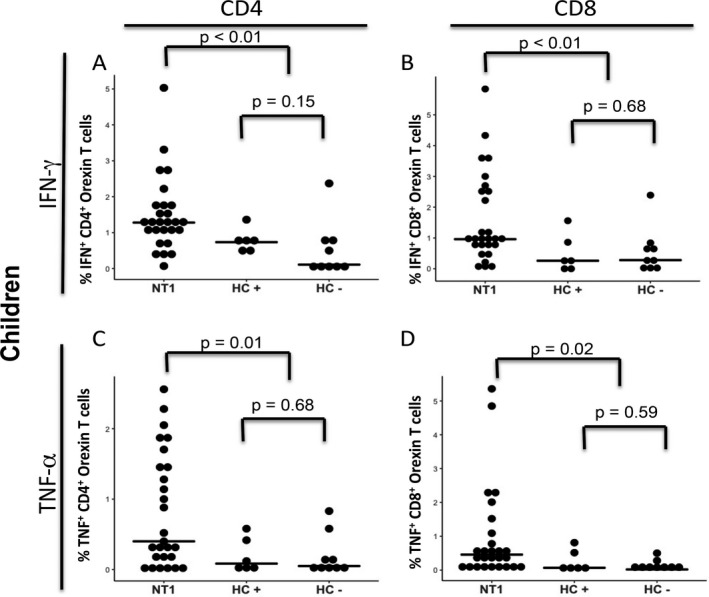
No difference in response to orexin between healthy control children with and without HLA‐DQB1*06:02 allele. Data from Figure 1 was further substratified by HLA‐DQB1*06:02 in healthy control children. Frequencies of CD4+ T‐cells (A, C) and CD8+ T‐cells (B, D) producing IFN‐γ (A, B) and TNF‐α (C, D) in response to orexin. NT1 = Narcolepsy Type 1, HC + = Healthy control children possessing the HLA‐DQB1*06:02 allele, HC ‐ = Healthy control children lacking the HLA‐DQB1*06:02 allele.

**Figure 3:**
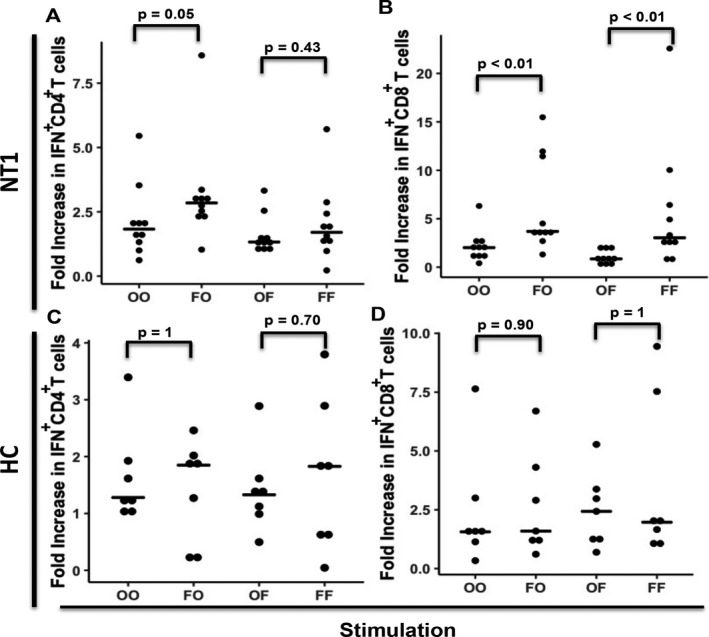
Priming with flu H1 increases the frequency CD4^+^ and CD8^+^ T‐cell response to orexins in NT1 but not HC children. PBMC from NT1 subjects (A, B) or HC (C, D) were initially stimulated with either an orexin (O) or the flu H1 protein (F) peptide library, and after 14 days cultured cells were restimulated with either the initial peptide library (OO, FF) or the reciprocal peptide library (OF, FO). The increase in the frequency of cells CD4^+^ or CD8^+^ T‐cells‐expressing IFN‐γ was calculated as a ratio of the respective response as compared to the baseline no peptide stimulation as described in Figure 1. The bars indicate the median results for the respective experimental conditions.

We used Rstudio for all statistical analysis and the software package ggplot2^15^ to generate all graphs. Student’s t test was used to compare the ages as well as the time from diagnosis to blood draw from the subjects. Mann–Whitney U tests were performed to compare NT1 and HC subjects in Figures 1, 2, and 3.

## Results

### Subject characteristics

Study subject characteristics are presented in Table 1. We recruited 41 patients diagnosed with NT1: 27 (66%) were children and 14 (34%) were adults. The average time from NT1 onset to sample collection was significantly shorter for children than for adults (3.3 years ± 2.11 vs. 11.4 ± 8.3; *P* < 0.001). All NT1 subjects were positive for the HLA DQB1*06:02 allele. In addition to the 41 NT1 subjects, we recruited 15 children and 16 adult healthy control (HC) subjects, who were of same age as the children and adult NT1 subjects. Of these individuals, 9/31(29%) were HLA DQB1*06:02 positive, with 6/15(40%) children and 3/16(19%) adults carrying the allele.

**Table 1:** Characteristics of subjects with NT1 and healthy controls

	Children NT1	Children HC	Adults NT1	Adults HC
n	27	15	14	16
Age range (y)	8‐21	8‐19	25‐63	22‐66
Avg age (y)	14.35±3.62	12.94±3.70	37±14.72	40±10.68
Sex	16 F / 11 M	10 F / 5 M	10 F / 4 M	7 F / 9 M
HLA‐DQB1*06:02^+^	27/27	6/15	14/14	3/16
Avg time from NT1 onset to blood draw (y)	3.3±2.1*		11.4±8.3	

Demographics of children and adults with Narcolepsy type 1 (NT1) and healthy controls (HC).

Abbreviation: Avg, Average.

* *P* < 0.001.

### Children but not adults with NT1 have a greater frequency of T‐cells responsive to orexin than healthy control subjects

Several reports have described the low frequency of orexin‐reactive T‐cells as an obstacle to their detection ex vivo.^9^, ^10^, ^11^ Therefore, we used a unique and sensitive gating strategy to detect those cells after extended stimulation with orexin peptides in vitro. A representative example of this ICS gating strategy to identify cytokine‐producing cells is shown in Figure 1A–B.

Using this gating strategy, we measured the percentage of T‐cells expressing IFN‐γ (Fig. 1C–F) in response to stimulation with orexin peptide library. Children with NT1 had a greater frequency of CD4^+^ and CD8^+^ T‐cells, cells that produced IFN‐γ than HC children (Fig. 1C and D). Interestingly, while there was a trend for more CD4^+^ T‐cells in the adult NT1 group, (Fig. 1E), it was not observed in the CD8^+^ T‐cells between adults with NT1 and HC (Fig 1F). In addition, we examined TNF‐α production after stimulation with the orexin peptide library, and observed similar results. Both CD4^+^ and CD8^+^ T‐cells had a higher frequency of TNF‐α^+^ cells in NT1 children compared to HC children (Fig. 1G and H). No such difference was observed in the adult NT1 and HC subjects in the frequency of TNF‐α‐producing CD4^+^ or CD8^+^ T‐cells (Fig. 1I and J). The frequency of orexin‐reactive T‐cells across the children and adult groups did not correlate with the time from NT1 onset (data not shown). In addition, there was no difference in T‐cell response to orexin peptides among healthy control children with and without the HLA‐DQB1*06:02 allele (Fig. 2).

### Flu priming increases the cellular immune response in children with NT1

As orexin‐specific T‐cells may be generated by viral molecular mimicry, we examined whether flu peptide priming amplifies the cellular immune response to orexin. We tested 10 NT1 children and 7 HC children; we first pulsed PBMCs with either Orexin (O) or Flu (F) peptide libraries to expand T‐cell populations, then rechallenged the cells with either the same library (OO, FF) or the reciprocal library(OF, FO), and compared the fold increase in the response from baseline between groups to examine cross‐reactivity. In children with NT1, priming with flu peptides significantly increased the response to orexin (FO) compared to orexin restimulation alone (OO) of both CD4^+^ (Fig. 2A) and CD8^+^(Fig. 2B) T‐cells. Interestingly, there was no difference in the ability of CD4^+^ T‐cells to respond to flu antigen after being primed by orexin (OF vs FF) (Fig. 2A), but CD8^+^ T‐cells showed a weaker response to flu antigens after being primed by orexin as compared to flu (OF < FF)(Fig. 2B). In contrast, HC children showed no difference in their ability to respond to orexin or flu antigen after being primed by either orexin or flu peptide libraries (Fig 2C and D).

## Discussion

In line with previous reports 9, 10, 11, we find a greater frequency of orexin‐reactive CD4^+^ and CD8^+^ T‐cells in people with NT1 compared to HC. The rarity of orexin‐reactive T‐cells has been a major obstacle for their detection in NT1. To circumvent this problem, Latorre et al,^9^ used clonal expansion and library screening in adults with NT1. Similarly, Luo et al^11^ and, Pedersen et al,^10^ resorted to a genetics‐based approach to identify orexin‐reactive T‐cells within their subjects. Unique to our findings is that we detected cytokine‐producing CD4^+^ and CD8^+^ T‐cells reactive to orexins in primary PBMC cultures. Furthermore, an increased frequency of these cells was found in children who, as a group, were closer to NT1 onset compared to adults, which suggests a decrease in orexin‐specific T‐cells circulating in blood over time This may explain our ability to directly identify orexin‐reactive T‐cells ex vivo as compared to other studies in which subjects were tested longer after NT1 symptoms onset. However, the lack of correlation between time from NT1 onset and frequency of orexin reactive T‐cells across the children and adult groups, suggests that differences in immune regulation between children and adults may also play an important role governing reactivity to orexin. Interestingly, while there was a trend toward a greater frequency of orexin‐reactive CD4^+^ T‐cells in adults with NT1 compared to adult HC, no such trend could be found in CD8^+^ T‐cells. Why orexin‐reactive CD8^+^ cells are rare in NT1 adults warrants further study.

Our results also suggest that priming with hemagglutinin increases the T‐cell response to orexin in children with NT1. This is consistent with the model that NT1 can be triggered by flu or flu vaccines.^4^ Interestingly, priming with orexin produced a similar response to rechallenge with flu compared to flu priming in CD4^+^ T‐cells. Why this occurs merits further study, as it may lead to a greater understanding of the cross–reactivity of orexin‐ and flu‐specific CD4^+^ populations.

Altogether, our data further indicate that CD4^+^ and CD8^+^ T‐cells autoreactive to orexins contribute to the development of NT1. Based on the preponderance of HLA DQB1:06:02, a MHC class II allele, in people with NT1, we had expected that this reactivity would be restricted to CD4^+^ T‐cells. However, the role of CD8^+^ T‐cells could be explained due to weaker associations between MHC‐I and NT1.^16^, ^17^ These findings are consistent with prior research using genetic^10^, ^11^ and clonal library^9^ approaches. One of the primary functions of CD8^+^ T‐cells is to lyse virally infected and damaged cells. It is therefore possible that an early viral infection can prime CD4^+^ and CD8^+^ T‐cells, causing increased orexin reactivity, leading to the destruction of the orexin‐producing neurons.

Our study is limited in that it is purely cross‐sectional and some patients were tested many years after symptoms onset**.** Prospective studies are needed to determine the dynamics of T‐cell reactivity against orexins over time in people with NT1, starting immediately at onset of symptoms, and to define the orexin epitopes recognized by CD4^+^ and CD8^+^ T‐cells, which may be involved in molecular mimicry in all stages of disease development. Altogether, our findings add credence to recent studies about the likely autoimmune pathology of NT1.^9^, ^10^, ^11^ This may have direct implications for the management of NT1, driven by early immunological testing closer to symptom onset to aid the clinical diagnosis and possible therapeutic interventions aimed at modulating both CD4^+^ and CD8^+^ T‐cell responses.

## Conflicts of interests

The authors have no potential conflicts of interest to report.

## Author contributions

AC and IJK contributed to the conception and design of the study; AC, DT, and ZO, contributed to the acquisition and analysis of the data; AC, KM, TES, and IJK contributed to drafting the text and preparing the figures.
